# Work–family conflict categories and support strategies for married female nurses: a latent profile analysis

**DOI:** 10.3389/fpubh.2024.1324147

**Published:** 2024-03-07

**Authors:** Xin Yao, Siqi Wen, Ziling Song, Jing Wang, Yuanyuan Shen, Xiaoqiong Huang

**Affiliations:** ^1^School of Ophthalmology and Optometry, Biomedical Engineering, Wenzhou Medical University, Wenzhou, Zhejiang, China; ^2^School of Nursing, Wenzhou Medical University, Wenzhou, Zhejiang, China; ^3^National Clinical Research Center for Ocular Diseases, Eye Hospital, Wenzhou Medical University, Wenzhou, Zhenjiang, China

**Keywords:** latent profile analysis, married female nurses, person-centered approach, work-family conflict, work-life balance, work-family border

## Abstract

**Objective:**

To clarify subgroups of married female nurses experiencing work–family conflict (WFC), explore the factors associated with the subgroups, and determine how desired support strategies differ among the subgroups.

**Methods:**

Data was collected from a sample of 646 married female nurses from public hospitals in Zhejiang Province, China, in December 2021. Latent profile analysis was used to group the participants, and multiple logistic regression was used to identify factors associated with higher WFC. The STROBE criteria were used to report results.

**Results:**

According to latent profile analysis, there were three distinct profiles of WFC among married female nurses: “low-conflict type,” “work-dominant-conflict type,” and “high-conflict type.” These profiles differed in the number of children, night shifts, family economic burden, childcare during working hours, family harmony, colleague support, and nurse–patient relationships. Nurses with multiple children, higher pressures in childcare during working hours, heavier family economic burdens, lower family harmony, lower colleague support, and poorer nurse–patient relationships are more likely to be classified as “high-conflict type” nurses.

**Conclusion:**

This study found that married female nurses experience different types of WFCs. The structure of these WFCs and their associated factors suggests that customized intervention strategies can be developed to address the specific needs of married female nurses.

## 1 Introduction

Work-life balance is an important topic in the field of occupational health psychology, particularly for women (1). Work-life balance has received less attention in Asia than in Western countries (2). Research has shown that conflict between work and family is one of the most significant sources of stress for working women (3, 4). The healthcare industry is rapidly growing, and its practitioners face numerous challenges related to work-life balance. According to previous survey results, 79.4% of nurses face challenges managing household affairs because of work demands, and 43.9% of them experience interruptions in work because of heavy family responsibilities (5). Additionally, female nurses face higher levels of work-life conflict than male nurses (6), and this phenomenon is more severe in nurses with young children (7). Therefore, married female nurses are highly vulnerable.

Currently, most countries are facing a shortage of nurses. The changing global population structure over the next 20 years will significantly increase the demand for healthcare services, further exacerbating nurse shortages (8). The primary reasons for the shortage of nurses include the declining proportion of young individuals in the workforce, the increasing proportion of older adult individuals, and the rising percentage of individuals aged 64 and above (8). Additionally, policy and planning barriers, barriers to training and enrolment, factors causing nursing staff turnover, and nurses’ stress and burnout contribute to nurse shortages (9). Several studies have demonstrated that increased work pressure and burnout among nurses contribute to high work–family conflict (WFC) (10, 11). In addition, WFC is an important factor leading to nurses’ turnover intention (12). Married female nurses constitute a considerable proportion of the nursing workforce and serve as the backbone of clinical nursing practices. The loss of this segment of human resources has implications for nursing quality, nursing workforce stability, hospital operational efficiency, and operating costs. Therefore, reducing WFC among married female nurses is an urgent concern for researchers and managers.

## 2 Background

The role stress arising from the incongruity between work and family demand is known as WFC (13). Fulfilling family responsibilities can become more difficult because of one’s work responsibilities, and vice versa (13). In hospitals, clinical nurses have diverse job duties and deal with numerous challenging and varied daily tasks. These tasks include clinical work (e.g., medication therapy, physical therapy, nursing planning, patient communication, health education), managing tasks (e.g., managing ward environment, inspecting medical equipment, coordinating bed and staff allocation), and enabling work (e.g., ward rounds, training of student nurses, departmental nursing education, assistance in conducting nursing research) (14). Within households, approximately three-quarters of the unpaid work worldwide is undertaken by women, amounting to a cumulative total of approximately 11 billion hours per day (15). Unpaid work encompasses services provided within the household to family members (16), such as housework, childcare, and personal care. A nationally representative family study from the UK revealed that during the initial COVID-19 pandemic, women performed about two-thirds of unpaid work and were more likely than men to reduce or adjust their work schedules due to increased time spent on unpaid work (17). The workload of nurses in different countries and the unpaid workload of married women is substantial, and the COVID-19 pandemic has undoubtedly exacerbated this phenomenon. In the context of the COVID-19 pandemic, married female nurses face significant pressure from their hospitals and families. However, research regarding the WFC experienced by this group remains limited. Given the distinct characteristics of this population, this study investigates and analyzes WFC experienced by married female nurses, aiming to provide insights that contribute to the enhancement of their occupational well-being.

Studying the WFC of nurses has significant theoretical and practical importance because nursing is a predominantly female profession carried out under demanding work conditions. Many studies have investigated the impact of WFC on patients, individuals, and organizations, with a particular focus on the nursing industry. Research conducted by Leodoro et al. revealed that higher levels of WFC were associated with a decline in nursing quality and an increase in adverse events (18), consequently affecting patient safety. Furthermore, high levels of WFC have substantial impacts on nurses’ health, increasing their risk of anxiety, depression (6), emotional exhaustion (19), sleep disorders (20), musculoskeletal pain (21), and cardiovascular risks (22). Moreover, WFC may lead to reduced work performance (23), job satisfaction (24), and organizational commitment (25) while simultaneously increasing job burnout (26) and turnover intentions (12). This can have negative effects on job conditions and organizational development. The adverse outcomes of WFC have drawn considerable attention from researchers, leading to the exploration of methods for reducing WFC and promoting work-family balance. Yildirim et al. discovered that social support can directly influence WFC (27), as it can reduce the occurrence of such conflicts by providing adequate organizational support and a conducive work-family balance environment (28). Orłowska et al. found that hospital management strategies based on nurses’ structural empowerment were beneficial for reducing WFC (29). Hospitals should create appropriate work environments that enable nurses to autonomously address the demands arising from work and family domains. Early research has identified numerous outcomes and influencing factors of WFC among nurses, but there has been relatively little focus on the subgroup of married female nurses. Previous research on WFC among nurses has predominantly used a variable-centered approach, focusing on the relationships between individual variables. However, this method cannot capture the comprehensive characteristics of WFC across its various dimensions. Latent profile analysis is a person-centered statistical method that identifies latent subgroups and their proportions based on individuals’ scores on observed variables (30), thus facilitating the examination of distinctive characteristics among different subgroups. This study aims to further explore distinct subtypes of WFC among married female nurses and investigate the characteristics and determinants of these subtypes. The goal is to provide valuable insights for the development of personalized intervention strategies.

Based on the research reviewed above, this study proposes the following research hypotheses: (1) WFC among married female nurses can be classified into distinct subtypes, (2) individual or work-related characteristics and support resources are associated with the subtypes of WFC among married female nurses, and (3) different subtypes of WFC among married female nurses require different support strategies. Validating these hypotheses can enhance our understanding of WFC among married female nurses, thus providing valuable insights for the development of targeted intervention measures and the promotion of nurses’ occupational health.

## 3 Methods

### 3.1 Aim

This study aimed to explore distinct subtypes of WFC experienced by married female nurses, identify potential factors associated with each subtype, and discuss the divergent supportive strategies required among these subtypes.

### 3.2 Design

This study was based on a self-reported cross-sectional research design, and a sample of 646 married female nurses in public hospitals in Zhejiang Province, China, was collected in December 2021. The study followed the STROBE guidelines for reporting cross-sectional studies (31).

### 3.3 Data collection

In November 2021, invitations were extended to tertiary and secondary hospitals located in Zhejiang, China to obtain preliminary authorization from hospital management personnel. Questionnaire links were distributed using convenience and snowball sampling methods. In December 2021, the participating nurses were initially provided with an anonymous online survey link via the Survey Star online platform to assess their willingness to participate. Subsequently, nurses who were willing to participate in the study and satisfied the prescribed inclusion criteria were invited to complete the questionnaire. To prevent survey duplication, each internet protocol address was restricted to a single questionnaire submission during survey completion. To ensure data integrity and address potential omissions, participants received prompts to review and supplement any missing information after clicking on the submission button, allowing for successful submission only after comprehensive input. Finally, data collection ceased if no new data uploads occurred within a seven-day period.

### 3.4 Participants

A total of 1,128 nurses were invited to participate, of whom 646 met the inclusion criteria and participated in the questionnaire survey. The inclusion criteria for the study population were as follows: (1) female gender; (2) married status; (3) age ≥ 18 years; (4) minimum work experience of 1 year; (5) registered nurse status. Nylund-Gibson et al. argued that a sample size within the range of 300–1,000 is generally considered the most commonly employed scope for effectively leveraging mixed-model fit indices (32). The sample size of this study adhered to statistical requirements.

### 3.5 Measures

#### 3.5.1 Demographic and work-related characteristics

Demographic and work-related characteristics of the participants were collected, encompassing variables such as age, education level, hospital level, years of clinical practice, professional title, employment type, annual income, health-related impacts on work, and job satisfaction. The assessment of the health-related impacts on work is operationalized through a binary question format: Does your current health status influence your work performance? 1 = Yes (indicating that the health status of married female nurses has certain effects on work, such as decreased work efficiency, increased absenteeism, or inability to complete work tasks), 2 = No (indicating that the health status of married female nurses does not affect work, such as work conditions unaffected by physical health status, or being able to maintain high efficiency even when occasionally feeling unwell). The job satisfaction of married female nurses is evaluated using the Likert four-point scale, as follows: How satisfied are you with your current job? Ranging from 1 = Satisfied to 4 = Not satisfied, with higher scores indicating lower job satisfaction.

#### 3.5.2 Family support resources

Family support resources were assessed through indicators such as the number of children, family financial pressure, and childcare stress at work. The number of children was used as an objective measure of familial caregiving pressure and was categorized into three distinct levels (1 = no children; 2 = one child; 3 = two or more children). Utilizing a binary yes-or-no format to assess family financial pressure, as illustrated below: currently, does your family face financial pressure? 1 = yes (indicating that your family encounters challenges in economic aspects, such as loans, daily expenses, and bills), 2 = no (denoting that your family is presently free from financial burdens). Employing a binary yes-or-no format to evaluate the childcare stress at work, as illustrated below: during your work hours, are you concerned about the care of your children? 1 = yes (signifying concerns about the availability or quality of childcare services during your work hours), 2 = no (indicating an absence of worries regarding childcare services during your work hours).

#### 3.5.3 Work support resources

Work support resources were primarily assessed through nurses’ perceived nurse–patient relationships and peer support.

Perceived support resources originating from patients were assessed using the Nurse–Patient Relationship Scale developed by Ma et al. (33). This scale comprises nine items scored on a six-point unipolar Likert scale according to the six-point Likert scoring method, ranging from 1 = strongly disagree to 6 = strongly agree. The items encompass two dimensions: nurse–patient trust and patient-centered care. Examples of the items include “Patients trust my nursing work,” “Patients believe I prioritize their care,” and “I strive to show care for patients during nursing work.” Total scores ranged from 9 to 54, with higher scores indicating more harmonious perceived nurse–patient relationships and a greater perception of patient-derived support resources. In Ma’s study, the content and construct validity were both confirmed (33). The Cronbach’s coefficient of the scale was 0.873, with coefficients of 0.900 and 0.883 for the two dimensions, signifying good internal consistency (33). In this study, the Cronbach’s coefficient for this scale was 0.960.

Perceived support resources from colleagues were evaluated using the Peer Support Scale developed by Ye (34). The scale comprised two components: Part A assessed the perceived support role of nurse managers, encompassing nine items, and Part B assessed the perceived support role of colleagues, consisting of 21 items. The scale is rated on a five-point Likert scale ranging from 1 = strongly disagree to 5 = strongly agree. Example items include “Nurse managers support me in seeking additional training or education for future development,” “The assistance provided by colleagues effectively alleviates my work stress,” and “Colleagues consistently acknowledge and demonstrate understanding of my emotions.” Total scores ranged from 30 to 150, with higher scores reflecting higher perceived colleague support from nurses. In Ye’s study, both A and B scales demonstrated good construct validity (34). Cronbach’s coefficients for Parts A and B were 0.922 and 0.959, respectively (34). In this study, the Cronbach’s coefficient for Part A was 0.970, while that for Part B was 0.986.

#### 3.5.4 Work–family conflict

The Work–Family Conflict and Family–Work Conflict Scales developed by Netemeyer et al. (35) were used to measure WFC. This scale encompasses 10 items scored on a five-point Likert scale ranging from 1 = strongly disagree to 5 = strongly agree. The items span two dimensions: work interference with family (WIF) and family interference with work (FIW). Example items include “The amount of time my job takes up makes it difficult to fulfill family responsibilities” and “I have to put off doing things at work because of demands on my time at home.” Total scores vary from 10 to 50, with higher scores indicating a greater severity of WFC experienced by nurses. In Netemeyer et al.’s study, the construct and discriminant validity were both confirmed (35). Cronbach’s coefficients for this scale range from 0.83 to 0.89 (35). In this study, the Cronbach’s coefficient for this scale was 0.900.

### 3.6 Statistical analysis

Latent Profile Analysis can be regarded as a person-centered approach aimed at identifying individual subgroups with similar score patterns across various indicators of interest (32, 36). In this study, Latent Profile Analysis was performed using Mplus 8.3 software. Using the scores from the 10 items of the Work–Family Conflict and Family–Work Conflict Scales as observed variables, one to five profiles were sequentially selected for analysis. The goodness of fit of the final model was assessed using the following three criteria (30). (1) Information evaluation criteria: Model fit was assessed by comparing the disparities between expected and actual values using the Akaike Information Criterion (AIC), Bayesian Information Criterion (BIC), and adjusted Bayesian Information Criterion (aBIC). A smaller statistical value indicated a better fit. (2) Classification evaluation criteria: The accuracy of classification was assessed using Entropy values, which range from 0 to 1. A value closer to one indicates a higher degree of classification accuracy. (3) Likelihood ratio tests: The fit differences between the models with k-1 and k profiles were compared using the Lo–Mendell–Rubin (LMR) and Bootstrap Likelihood Ratio test (BLRT). When the *p*-value for the LMR and BLRT was less than 0.05, a model with k profiles was superior to a model with k-1 profiles. The aforementioned evaluation criteria were intended as a reference for profile decision making. When determining an optimal model, it is important to consider the interpretability of each category.

Data analysis was conducted using SPSS 27.0 statistical software. The statistical methods employed encompassed descriptive statistical calculations (means, standard deviations, percentages), one-way analysis of variance, chi-square (χ^2^) tests, rank-sum tests, and multinomial logistic regression analysis. The significance level was set at *α* = 0.05, with *p* < 0.05 indicating statistically significant differences.

### 3.7 Ethical considerations

This study was approved by the ethics committees of the Eye Hospital of Wenzhou Medical University on May 25, 2020 (no. 2020-J-68). The introductory section of the electronic questionnaire presented relevant study information to all participants, including the research background, study objectives, research methodology, information confidentiality, voluntary participation, and the option to withdraw. Following the introduction, those who agreed to participate in the study completed the questionnaire. All data collected from the survey were kept anonymous and access to this information was restricted to the members of the research team.

## 4 Results

### 4.1 Participant characteristics

The study included 646 participants, all of whom were married female nursing professionals in Zhejiang. The participants’ mean and standard deviation for age, years of clinical practice, and annual income were 34.12 ± 6.43, 12.74 ± 7.05, and 100.92 ± 53.19, respectively. Most participants had a bachelor’s degree (76.0%), were nurse practitioners (62.5%), were affiliated with tertiary hospitals (70.0%), were contract-based nurses (59.4%), had one child (61.1%), experienced financial pressure within their families (61.9%), reported no health-related impacts on work (83.6%), expressed concerns about childcare while working (76.9%), and had a moderate level of job satisfaction (62.2%). The nurses’ demographics are presented in Table 1.

**Table 1:** Demographic characteristics of nurses and their single-factor analysis on the work–family conflict profile.

Variables		Overall (*n* = 646)	Low-conflict type(*n* = 201)	Work-dominant- conflict type(*n* = 208)	High-conflict type (*n* = 237)	Test statistics	*p*-value
		M ± SD	M ± SD	M ± SD	M ± SD		
Age		34.12 ± 6.43	34.13 ± 6.68	34.61 ± 6.59	33.68 ± 6.06	1.151^3^	0.317
Years of clinical practice		12.74 ± 7.05	12.93 ± 7.39	13.49 ± 7.25	11.93 ± 6.50	2.850^3^	0.059
Annual income, thousand/y (RMB)		100.92 ± 53.19	98.23 ± 59.47	105.79 ± 49.06	98.93 ± 50.92	1.295^3^	0.275
Education level	Below associate degree	5(0.8%)	2(1.0%)	1(0.5%)	2(0.8%)	4.931^2^	0.085
	Associate degree	146(22.6%)	56(27.9%)	43(20.7%)	47(19.8%)		
	Bachelor’s degree	491(76.0%)	142(70.6%)	161(77.4%)	188(79.3%)		
	Master’s degree and above	4(0.6%)	1(0.5%)	3(1.4%)	–		
Professional title	Nurse practitioner	404(62.5%)	117(58.2%)	131(63.0%)	156(65.8%)	3.669^1^	0.721
	Nursing team leader	111(17.2%)	37(18.4%)	38(18.3%)	36(15.2%)		
	Supervisor nurse	117(18.1%)	41(20.4%)	35(16.8%)	41(17.3%)		
	Nursing department director	14(2.2%)	6(3.0%)	4(1.9%)	4(1.7%)		
Hospital level	Secondary hospital	194(30.0%)	64(31.8%)	62(29.8%)	68(28.7%)	0.520^2^	0.771
	Tertiary hospital	452(70.0%)	137(68.2%)	146(70.2%)	169(71.3%)		
Employment type	Bianzhi nurses	262(40.6%)	69(34.3%)	94(45.2%)	99(41.8%)	5.233^1^	0.073
	Contract-based nurses	384(59.4%)	132(65.7%)	114(54.8%)	138(58.2%)		
Number of children	0	78(12.1%)	33(16.4%)	24(11.5%)	21(8.9%)	11.609^2^	0.003
	1	395(61.1%)	129(64.2%)	124(59.6%)	142(59.9%)		
	≥2	173(26.8%)	39(19.4%)	60(28.8%)	74(31.2%)		
Family financial pressure	Yes	400(61.9%)	95(47.3%)	126(60.6%)	179(75.5%)	37.081^1^	<0.001
	No	246(38.1%)	106(52.7%)	82(39.4%)	58(24.5%)		
Health-related impacts on work	Yes	106(16.4%)	12(6.0%)	32(15.4%)	62(26.2%)	32.558^1^	<0.001
	No	540(83.6%)	189(94.0%)	176(84.6%)	175(73.8%)		
Childcare stress at work	Yes	497(76.9%)	129(64.2%)	163(78.4%)	205(86.5%)	30.884^1^	<0.001
	No	149(23.1%)	72(35.8%)	45(21.6%)	32(13.5%)		
Job satisfaction	Very satisfied	179(27.7%)	104(51.7%)	46(22.1%)	29(12.2%)	90.195^2^	<0.001
	Moderately satisfied	402(62.2%)	91(45.3%)	138(66.3%)	173(73.0%)		
	Slightly dissatisfied	50(7.7%)	4(2.0%)	19(9.1%)	27(11.4%)		
	Not satisfied	15(2.3%)	2(1.0%)	5(2.4%)	8(3.4%)		

M, mean; SD, standard deviation.^1^Chi-square test; ^2^rank-sum test; ^3^one-way analysis of variance.

### 4.2 Latent profile model selection results

Initially, five latent profile models were fitted (Table 2). As the number of extracted latent profiles increased from one to three, the AIC, BIC, and aBIC decreased. The LMR and BLRT results were statistically significant (*p* < 0.05). Each nurse category was assigned to a latent profile with an average probability ranging from 0.962 to 0.979%, indicating a high level of credibility for the three latent profile models. However, when the number of extracted latent profiles increased from three to four or five, the LMR test did not reach significance (*p* = 0.7819 and *p* = 0.2467, respectively). After a comprehensive analysis, we retained three latent profiles.

**Table 2:** Model fitting information for the latent profile of nurses’ work–family conflict.

Model	AIC	BIC	aBIC	Entropy	*p-*value		Class probability(%)
					LMR	BLRT	
1	31803.583	31919.824	31837.275	–	–	–	–
2	27975.799	28154.631	28027.633	0.956	<0.001	<0.001	0.44/0.56
3	26383.360	26624.784	26453.336	0.940	0.0156	<0.001	0.31/0.32/0.37
4	25525.401	25829.416	25613.518	0.940	0.7819	<0.001	0.32/0.29/0.29/0.11
5	24442.627	24809.233	24548.886	0.957	0.2467	<0.001	0.19/0.18/0.27/0.26/0.10

AIC, Akaike Information Criterion; BIC, Bayesian Information Criterion; aBIC, adjusted Bayesian Information Criterion; LMR, Lo–Mendell–Rubin; BLRT, Bootstrap Likelihood Ratio test.

**Figure 1:**
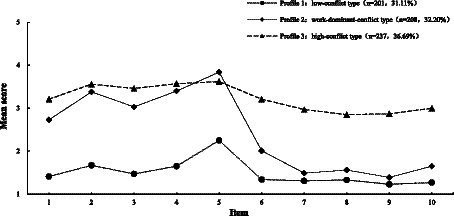
Characteristics of latent profile in work–family conflict of nurses. Latent class analysis divided the population into three groups: low-conflict type, work-dominant**-**conflict type, and high-conflict type. The line graph shows how high or low each group scored on the 10 items of the Work–Family Conflict and Family–Work Conflict Scales, representing each type of feature.

Based on the nurses’ performance on the Work–Family Conflict and Family–Work Conflict Scales, categories 1–3 were labeled. The WIF dimension scores for married female nurses were 14.39 ± 4.91 points and the FIW dimension scores were 10.15 ± 4.35 points. Figure 1 illustrates the scores of categories 1–3 for each item of the Work–Family Conflict and Family–Work Conflict Scales. Nurses in category 1 were labeled as “low-conflict type” due to their relatively low scores in the WIF dimension (8.60 ± 2.57 points) and the FIW dimension (6.55 ± 1.95 points). This group comprised 201 nurses, accounting for 31.11% of the total sample. Nurses in category 2 were termed “work-dominant-conflict type” as they exhibited higher scores in the WIF dimension (16.58 ± 2.94 points) but lower scores in the FIW dimension (8.15 ± 2.09 points). This group comprised 208 nurses, representing 32.20% of the total population. Nurses in category 3, designated as the “high-conflict type,” scored at elevated levels in the WIF dimension (17.40 ± 3.31 points) and the FIW dimension (14.96 ± 2.61 points). This group comprised 237 nurses, accounting for 36.69% of the total population.

### 4.3 Characteristics of nurses’ work–family conflict latent profiles

Tables 1, 3 present the descriptive characteristics of Classes 1–3, along with the results of the intergroup difference tests indicated by *p*-values. Significantly different profiles were observed among the three classes regarding factors such as the number of children, family financial pressure, health-related impacts on work, childcare stress at work, job satisfaction, nurse–patient relationships, nurse manager support, and colleague support *(p* < 0.05). However, there were no significant differences in age, years of clinical practice, education level, professional title, hospital level, employment type, and annual income (*p* > 0.05).

**Table 3:** Comparison of scores of scale between different latent profiles.

Variables	Dimensions	Overall	Low-conflict type(*n* = 201)	Work-dominant-conflict type(*n* = 208)	High-conflict type (*n* = 237)	*F*	*p*-value
		M ± SD	M ± SD	M ± SD	M ± SD		
NPRS	Nurse–patient trust	19.15 ± 3.47	20.26 ± 3.76	1.71 ± 3.25	17.73 ± 2.87	36.529	<0.001
	Patient-centered care	25.60 ± 4.23	26.85 ± 4.39	26.48 ± 3.61	23.76 ± 3.96	39.884	<0.001
	Total	44.75 ± 7.27	47.10 ± 7.79	46.19 ± 6.26	41.49 ± 6.43	43.471	<0.001
PSS	Nurse manager support	33.11 ± 7.17	35.06 ± 7.95	32.91 ± 7.27	31.63 ± 5.93	13.047	<0.001
	Colleague support	78.36 ± 15.10	83.55 ± 16.70	79.20 ± 14.15	73.22 ± 12.70	28.062	<0.001
	Total	111.47 ± 20.82	118.61 ± 23.40	112.11 ± 19.47	104.85 ± 17.32	25.713	<0.001

M, mean; SD, standard deviation; NPRS, Nurse–Patient Relationship Scale; PSS, Peer Support Scale.

Among these three categories, the characteristics of “low-conflict type” nurses and “high-conflict type” nurses were the most distinct. “Low-conflict type” nurses are most likely to have no children or only one child, experience lower family financial pressure, have a better health status, encounter fewer childcare stressors while working, and experience higher job satisfaction. Additionally, this group of nurses scored higher in nurse–patient relationships, perceived nurse manager support, and colleague support. “High-conflict type” nurses are more likely to have multiple children, bear heavier family financial pressures, experience poorer health status, worry about childcare stress while working, and have lower job satisfaction. Likewise, this group of nurses scored lower in nurse–patient relationships, perceived nurse manager support, and colleague support. “Work-dominant-conflict type” nurses did not exhibit distinctive characteristics in other aspects.

### 4.4 Predictive factors of nurses’ work–family conflict latent profiles

Using the variables that showed statistical significance (*p* < 0.05) in the analysis of variance as independent variables, a multinomial logistic regression analysis was conducted with the latent profiles of nurses’ WFC as the dependent variable. The results of the logistic regression analysis revealed that colleague support, nurse manager support, nurse–patient relationships, job satisfaction, number of children, childcare stress at work, health-related impacts on work, and family financial pressure were influential factors for the latent profiles of “high-conflict type” nurses in WFC. Job satisfaction, number of children, childcare stress at work, and health-related impacts on work are influential factors for the latent profile of “work-dominant-conflict type” nurses in WFC (see Table 4 for details).

**Table 4:** Multi-factor analysis of three latent profiles in work–family conflict of nurses.

Variables	High-conflict type (vs. Low-conflict type)					Work-dominant-conflict type (vs. Low-conflict type)				
	*Β*	Wald test	*p-*value	Odds ratio	95% confidence interval	*Β*	Wald test	*p-*value	Odds ratio	95% confidence interval
PSS										
Colleague support	−0.038	10.517	0.001	0.96	0.94 ~ 0.99	−0.010	0.886	0.347	0.99	0.97 ~ 1.01
Nurse manager support	0.052	4.489	0.034	1.05	1.00 ~ 1.11	−0.003	0.018	0.892	1.00	0.96 ~ 1.04
NPRS	−0.087	19.628	<0.001	0.92	0.88 ~ 0.95	0.010	0.265	0.606	1.01	0.97 ~ 1.05
Job satisfaction	1.222	31.380	<0.001	3.39	2.21 ~ 5.20	1.070	26.796	<0.001	2.92	1.95 ~ 4.37
Number of children										
1	1.005	8.064	0.005	2.73	1.37 ~ 5.47	0.536	2.741	0.098	1.71	0.91 ~ 3.22
≥2	1.700	18.114	<0.001	5.47	2.50 ~ 11.97	1.088	8.757	0.003	2.97	1.44 ~ 6.10
0^a^										
Childcare stress at work	1.092	14.731	<0.001	2.98	1.71 ~ 5.21	0.662	7.022	0.008	1.94	1.19 ~ 3.16
Health-related impacts on work	1.392	13.990	<0.001	4.02	1.94 ~ 8.34	0.847	5.187	0.023	2.33	1.13 ~ 4.84
Family financial pressure	0.608	6.388	0.011	1.84	1.15 ~ 2.94	0.111	0.254	0.614	1.12	0.73 ~ 1.72

PSS, Peer Support Scale; NPRS, Nurse–Patient Relationship Scale. ^a^Reference category.

## 5 Discussion

To the best of our knowledge, this study represents the first investigation of the WFC profiles of married female nurses. Our findings revealed three profiles distinguished by their different structural combinations. We identified the predictive factors for nurses with high levels of conflict and explored customized intervention strategies that could enhance the occupational well-being of married female nurses. Based on the findings of this study, we are prepared to engage in comprehensive discussions aimed at deepening our understanding of these research questions.

Based on the findings of this study, we observed that WIF was greater than FIW in the context of WFC among married female nurses. This result aligns with previous research, which indicates that the work responsibilities of married female nurses have a greater impact on their family obligations than the influence of family responsibilities on their work duties (11, 20, 37). While researchers have explored the reasons behind conflict arising within the dimensions of work and family, that is, how the domains mutually influence each other and lead to conflict, there has been limited investigation into why work-related conflict tends to surpass family-related conflict, that is, the reasons for WIF being greater than FIW. Several factors can contribute to this phenomenon, spanning various levels. At the societal level, the COVID-19 pandemic caused medical supply shortages and exposed nurses to infection risks, and thus increased nurses’ work-related stress (38). In addition, advancements in communication technology have made it easier for supervisors to contact employees outside of working hours, which further depletes nurses’ personal time (39). At the organizational level, hospitals are environments with strict disciplines, and China’s collectivist culture emphasizes group interests over individual interests, leading nurses to comply with organizational decisions when their personal lives are disrupted by work (40), intensifying WFC. At the family level, handling family affairs often allows for more flexibility regarding time and approach compared to managing work-related issues. Family members tend to be more understanding and tolerant of one another, resulting in less interference with work. At the individual level, medical-related work often involves significant interests, and nursing operates under a system of responsibility. Even in the majority of cases where the workload becomes overwhelming, making it difficult for nurses to complete their tasks within designated hours, leaders and the work environment do not tolerate nurses leaving unfinished work or going off duty. Consequently, nurses frequently engage in unpaid overtime, which results in extended working hours.

This study’s findings reveal the types of WFC experienced by married female nurses during the COVID-19 period. Based on latent profile analysis results, three types of WFC have been identified: “low-conflict type” (31.11%), “work-dominant-conflict type” (32.20%), and “high-conflict type” (36.69%). These results are consistent with previous research by Vaziri et al. (37), Zhang et al. (41), Rantanen et al. (42), and other research teams that investigated employees from various industries using the Work–Family Conflict and Enrichment Questionnaires, demonstrating the heterogeneity of the Work-Family Interface types. While the Work-Family Interface exhibits overall heterogeneity, its classification with respect to WFC is consistent, and WFC can be categorized into three types: high in WIF and FIW, higher in WIF than in FIW, and low in WIF and FIW. Border theory is a theory in the work-family domain (43) that offers a concrete theoretical framework to explain phenomena in the work-family field and the reasons behind WFC classification. According to this theory, individuals are boundary spanners, transitioning between work and family every day (44). Boundaries, which can be physical, temporal, or psychological, exist between work and family domains. These boundaries may protect the respective domains, but they are not impermeable, allowing for information, emotions, and behaviors to flow across different boundaries. Nurses classified as “low-conflict type” effectively differentiate their work and family roles, establishing relatively impermeable boundaries between the two domains. This separation enables them to maintain relative independence between their work and family realms, thereby preventing mutual interference. Nurses classified as the “work-dominant-conflict type,” while capable of distinguishing between work and family domains, may encounter conflicts in effectively managing these two realms when burdened with substantial work responsibilities. The demands inherent in their professional roles could significantly encroach on their family roles, resulting in conflict. In the Chinese cultural context, where family is of paramount importance in individuals’ lives, there is a strong emphasis on family stability, cohesion, and overall well-being. The traditional Chinese family system leans toward conservatism, highlighting the importance of individuals fulfilling their roles and obligations within the family structure (45). Consequently, within a cultural backdrop centered around the family and characterized by prevalent traditional gender roles, nurses not only grapple with the demands of their professional roles but also strive to meet familial expectations. This dual expectation dynamic gives rise to conflicts as nurses navigate the intricate balance between their professional and familial commitments. Nurses classified as “high-conflict type” face higher levels of conflict in the work and family domains. This clearly reflects the characteristics of boundary intensity, where stronger permeability, flexibility, and blending of boundaries indicate weaker boundary intensity, making WFC more likely to occur. Based on the above results, addressing WFC among married female nurses can be approached from two perspectives. For “work-dominant-conflict type” nurses, the best strategy is to reduce their work stress and promote a supportive work culture. For “high-conflict type” nurses, alleviating WFC can be achieved by strengthening their boundary intensity.

Social support is one of the most extensively studied antecedents of WFC (46). Social support refers to beneficial behaviors such as caring, giving advice, lending a helping hand, and providing relevant feedback (47). Considerable discussion has centered on the specific role of social support in reducing conflicts between work and family (48). Social support can arise from work or family domains (46). This study found that work and family support resources are associated with the type of WFC experienced by married female nurses. Specifically, married female nurses who have fewer support resources from patients, supervisors, colleagues, and families are more likely to be classified as “high-conflict type” nurses. This result is consistent with multiple meta-analyses (46) showing that social support from work and family domains can reduce employees’ WFC. Perceived support from supervisors and colleagues as well as good communication with patients can help nurses alleviate stress, especially for married female nurses under dual pressure from work and family, and reduce conflict between work and family. Nurses classified as “low-conflict type” effectively differentiate their work and family roles, establishing relatively impermeable boundaries between the two domains. Thus, work support resources are most likely to relieve nurses’ stress when they face high job pressure because supervisors, colleagues, and patients can provide the resources needed to deal with such work stress. Organizational interventions should focus on building nurses’ informal networks with patients, leaders, and colleagues to strengthen cooperation and mutual support. Family support resources are crucial for married female nurses. A lack of family support resources directly leads to WFC (23, 49). Married women undertake more household and childcare duties than men (50). When married female nurses cannot obtain reliable childcare resources, adequate family finances, or harmonious family relationships, they may find it difficult to balance work and family, thus adding stress to their lives. Many family support resources can help married female nurses reduce WFC, including adopting flexible work arrangements (51), formulating family friendly work policies (52), providing adequate spousal support (53), and providing social support through multimedia educational programs (54).

The proportion of “high-conflict type” or “work-dominant-conflict type” nurses is relatively high, with approximately 70% of married female nurses belonging to these categories. These two groups have distinct characteristics, thus necessitating the proposal of different support strategies to reduce their WFC. “High-conflict type” nurses are characterized by weak boundary strength and limited work-family support resources. From an individual perspective, Clark’s (44) boundary management can be used to develop strategies for mitigating WFC. For example, nurses can strengthen physical boundaries by reducing their use of electronic communication devices outside of work hours, strengthen temporal boundaries by improving their control over their schedules, and strengthen psychological boundaries by guiding their behavior patterns (e.g., minimizing work-related tasks during work hours and vice versa). From an organizational perspective, researchers should conduct qualitative inquiries into work–family conflict, identifying instances and the severity of boundary violations among married female nurses. Additionally, advocacy is warranted for a shift in focus from individual responsibility to organizational responsibility, minimizing additional burdens on married female nurses. Systemic interventions at the institutional level should be considered for work-induced boundary violations, such as establishing flexible employment mechanisms and providing support services for work and family life. For family-induced boundary violations, addressing societal support mechanisms for women is crucial. This may involve fostering a culture that promotes work-life balance, challenging traditional gender role perceptions, and refining social security systems related to parenting services. Such multi-faceted interventions can provide a more sustainable and equitable environment for nurses. “Work-dominant-conflict type” nurses are characterized by excessive work pressure. This can be alleviated using novel technologies, such as artificial intelligence (AI). The rapid development of AI, driven by substantial technological investments and government support initiatives, has significantly affected various industries, including nursing. In a clinical setting, AI technology can assume tasks traditionally performed by nurses (such as writing nursing documentation and devising care plans) (55), altering the manner in which nurses provide care to patients and maximizing the amount of time spent on patients. The future envisions utilizing AI to assist nurses in their caregiving responsibilities, thereby mitigating WFC.

## 6 Limitations and future research

This study has certain limitations that should be addressed in future research. First, it adopted a cross-sectional research design, which rendered it incapable of drawing conclusions regarding causal relationships. Currently, research on nurse WFC predominantly relies on cross-sectional studies, with fewer instances of qualitative research, longitudinal studies, and intervention studies. Therefore, in the future, one can employ a variety of research methodologies to explore nurse WFC to enhance the richness of its outcomes. Additionally, only a limited number of interventional studies aimed at mitigating WFC have been published. In the future, scholars should conduct more experimental research based on existing theoretical investigations to validate and generalize the findings of theoretical research. Second, this study exclusively encompasses hospitals within Zhejiang province, China, thereby lacking generalizability across other regions within China. Hence, future research should encompass a wider range of provinces and countries to corroborate the findings of this study. Third, this study utilized convenience and snowball sampling. Convenience sampling may introduce selection bias, and snowball sampling may exacerbate this bias and lack randomness. Consequently, the external validity and generalizability of study results to a broader population may be compromised. Fourth, this study relied on a self-report questionnaire to collect data. However, self-reporting can be vulnerable to potential biases such as recall and social desirability biases. These biases may be particularly relevant for nurses, given their high rates of professional burnout and elevated stress levels. Considering these limitations, future research would benefit from exploring the use of objective measurement methods, such as physiological markers or work-related metrics, to assess nurses’ work–family conflict. While these methods may present their own challenges in terms of cost or feasibility, they could potentially provide more accurate and reliable results. Fifth, in this study, rather than demographic and work-related characteristics among married female nurses, it was shown that factors relating to family and work support resources have a greater influence on the typology of WFC. This study’s results showed that family and work support resources could predict “high-conflict type” nurses, while among demographic and job characteristics, only health-related impacts on work and job satisfaction could predict the types. This may be because the mechanisms through which demographic and job characteristics influence WFC are more complex and there are often interaction effects among these factors, obscuring their impact on WFC types. Sixth, the selected survey indicators failed to effectively distinguish between “low-conflict type” and “work-dominant-conflict type” nurses. “Work-dominant-conflict type” nurses accounted for a large proportion (32.20%) of the sample. Follow-up studies need to continue exploring the characteristics of such nurses, identifying their inherent mechanisms, and proposing targeted strategies to reduce WFC.

## 7 Conclusion

This study revealed that married female nurses experience a greater degree of WIF than FIW. In this group, WFC can be categorized into three subtypes: “low-conflict type,” “work-dominant-conflict type,” and “high-conflict type.” Additionally, the availability of work-related and family-related support resources is associated with the categorization of potential profiles among married female nurses. For those falling under the “high-conflict type,” leveraging the social support system is crucial to help establish clear boundaries between work and family domains. For the “work-dominant-conflict type,” future initiatives may explore strategic investments in the development and implementation of AI technologies to alleviate the workload of married female nurses and mitigate work-related stress. In summary, mitigating WFC among married female nurses is crucial. To effectively address this conflict, it is essential to consider the specific categories of WFC that nurses face and develop targeted support strategies accordingly. In future research endeavors, it is advisable to further explore the influencing factors that impact the types of WFC experienced by married female nurses. Additionally, conducting more intervention studies to apply and validate theoretical findings in the realm of WFC would be beneficial.

## Data availability statement

The data analyzed in this study is subject to the following licenses/restrictions: our research team is currently utilizing this dataset for ongoing studies, temporarily restricting its public availability. Requests to access these datasets should be directed to yaoxin2021yao@163.com.

## Ethics statement

The studies involving humans were approved by the ethics committees of the Eye Hospital of Wenzhou Medical University. The studies were conducted in accordance with the local legislation and institutional requirements. The participants provided their written informed consent to participate in this study.

## Author contributions

XY: Writing – original draft, Writing – review & editing, Conceptualization, Data curation, Formal analysis, Investigation, Methodology, Software, Validation. SW: Conceptualization, Data curation, Investigation, Writing – review & editing. ZS: Data curation, Investigation, Supervision, Writing – review & editing. JW: Data curation, Investigation, Supervision, Writing – review & editing. YS: Data curation, Investigation, Supervision, Writing – review & editing. XH: Conceptualization, Project administration, Resources, Supervision, Writing – original draft, Writing – review & editing.
